# Cardiac fibrosis induced by high‐fat diet in ApoE‐deficient male mice is exacerbated by genetic deletion of PACAP–PAC1 signaling

**DOI:** 10.1111/jne.70118

**Published:** 2025-11-25

**Authors:** J. M. Schubart, M. K. H. Schaefer, G. A. Bonaterra, L. Mey, H. Schwarzbach, S. Pankuweit, F. Ausbuettel, L. E. Eiden, S. Weyand, E. Weihe, R. Kinscherf, C. Waechter

**Affiliations:** ^1^ Department of Medical Cell Biology, Institute for Anatomy and Cell Biology, Medical Faculty Philipps University Marburg Marburg Germany; ^2^ Center for Mind, Brain and Behavior Philipps University Marburg Marburg Germany; ^3^ Department of Cardiology, University Hospital Marburg Philipps University Marburg Marburg Germany; ^4^ Section on Molecular Neuroscience, Laboratory of Cellular and Molecular Regulation National Institute of Mental Health Intramural Research Program Bethesda Maryland USA; ^5^ Department of Cardiology Ostalb Clinic Aalen Germany

**Keywords:** antifibrotic effect, cardio protection, HFpEF, PAC1, PACAP

## Abstract

Cardiac fibrosis is characterized by an excessive accumulation of extracellular matrix proteins and occurs in a variety of cardiac diseases, such as the highly prevalent syndrome heart failure with preserved ejection fraction (HFpEF) and other cardiac disorders. Interstitial fibrosis has been identified as a central pathophysiological factor induced and maintained by metabolic stress and chronic inflammation. Considering the limited treatment options for cardiac fibrosis, new therapeutic targets are urgently needed. Mounting evidence for the cardioprotective effects of the neuropeptide pituitary adenylate cyclase‐activating peptide (PACAP) provides a rationale to elucidate its role and that of its receptor PAC1 in metabolic stress‐mediated cardiac fibrosis. Metabolic stress was induced by feeding a cholesterol‐enriched diet (CED) to PACAP^−/−^/ApoE^−/−^, PAC1^−/−^/ApoE^−/−^ and ApoE^−/−^ mice and cardiac tissue subjected to analyses of fibrosis. Under CED feeding, a statistically significant (*p* < .001) increase in myocardial fibrosis was observed in PACAP^−/−^/ApoE^−/−^ and PAC1^−/−^/ApoE^−/−^ compared to ApoE^−/−^ mice. These findings suggest a role for PACAP signaling in the mitigation of metabolically induced cardiac fibrosis. The antifibrotic effect of PACAP is dependent on the expression of the PAC1 receptor and only emerges under metabolic stress conditions. PAC1 receptor agonists may have the potential to attenuate metabolically triggered cardiac fibrosis arising after a chronic high‐fat diet.

## 1 INTRODUCTION

Fibrosis is a hallmark of cardiac remodeling and is present in a wide variety of cardiac diseases.^1^ Reparative fibrosis occurs, for example, after myocardial infarction to preserve the structural integrity of the heart and thus prevent fatal cardiac rupture. In addition, there is cardiac fibrosis that primarily affects the interstitium.^2^ This type of cardiac fibrosis develops insidiously without loss of cardiomyocytes and is characterized by interstitial and perivascular deposition of extracellular matrix (ECM) proteins.^3^ As a result, myocardial stiffness increases and ventricular compliance decreases, leading to diastolic dysfunction. Interstitial fibrosis is a pivotal factor contributing to heart failure, for example, in the syndrome of heart failure with preserved ejection fraction (HFpEF).^4^ Current evidence suggests that comorbidities, predominantly from the metabolic spectrum, such as obesity, *diabetes mellitus* and arterial hypertension, are potent drivers of chronic systemic low‐grade inflammation and constitute a cardinal pro‐fibrotic stimulus in many cardiac diseases including HFpEF.^5^, ^6^ Whereas several prognosis‐improving pharmacological therapies are available for heart failure in which reparative fibrosis (e.g., after myocardial ischemia) plays a central role, the therapeutic options for non‐reparative/interstitial fibrosis (e.g., associated with the metabolic syndrome) are very limited and new options are urgently needed, given the high prevalence of related morbidities.^7^

A growing body of evidence for cardioprotective effects of the neuropeptide pituitary adenylate cyclase‐activating peptide (PACAP) renders it a promising candidate for further research in this area. PACAP belongs to the vasoactive intestinal polypeptide (VIP)/secretin/somatoliberin/glucagon superfamily and exhibits evolutionary conservation with complete homology among mammalian species.^8^ The peptide is present in two isoforms: PACAP‐27 and PACAP‐38, which are 27 or 38 amino acid residues in length, respectively, and exerts pleiotropic functions mediated by 3 G‐protein‐coupled receptors, which are preferentially associated with activation of adenylate cyclase: VPAC1, VPAC2, and PAC1.^9^ Whereas VPAC1 and VPAC2 receptors show a comparable high affinity for both VIP and PACAP, the PAC1 receptor binds PACAP with high affinity and VIP with much lower affinity.^9^, ^10^ In the clinical context, PACAP has previously attracted attention because of its role as a potent trigger of migraine headaches, which has led to the exploration of strategies to antagonize the PACAP–PAC1 pathway as a potential new therapeutic approach for this significant neurological disorder.^8^, ^11^, ^12^ However, in support of the pleiotropic nature of PACAP, a broad spectrum of favorable effects on various organs and tissues has also been demonstrated. For example, in the cardiovascular context, PACAP activation of the PAC1 receptor has been shown to inhibit protein synthesis in cardiac fibroblasts and to stimulate the release of atrial natriuretic peptide (ANP) in rat cardiomyocytes in vitro, thus potentially exerting anti‐fibrotic effects.^13^ In vivo models of cardiotoxicity involving doxorubicin or mitoxantrone administration or irradiation also revealed anti‐fibrotic as well as anti‐inflammatory and anti‐apoptotic effects of PACAP.^14^, ^15^, ^16^ The apolipoprotein E knockout (ApoE^−/−^) mouse is an established model for studying atherogenesis, and also exhibits a marked systemic inflammatory and pro‐fibrotic status in conjunction with a Western‐type cholesterol‐enriched diet (CED). In addition, we have previously shown that the PAC1 receptor agonist maxadilan attenuates atherosclerosis in ApoE^−/−^ mice fed a high‐fat diet.^17^ This model has therefore been chosen here to examine the possible involvement of PACAP signaling through PAC1, in the pathogenesis of metabolic stress‐induced cardiac fibrosis.^18^, ^19^

## 2 METHODS

### 2.1 Animals

C57BL/6 mice with ApoE knockout (ApoE^−/−^) (Charles River, Sulzfeld, Germany) were crossbred with PACAP‐ or with PAC1‐deficient mice to generate PACAP^−/−^ApoE^−/−^ and PAC1^−/−^ApoE^−/−^ genotypes. PACAP (Adcyap1) and PAC1 (PAC1R, Adcyap1r1) knockout mice were generated as previously described.^20^, ^21^ After 10 weeks of standard chow diet (SD) (LASQCdiet® Rod16 Rad; LASvendi, Soest, Germany) male mice homozygous for PACAP^−/−^/ApoE^−/−^ or PAC1^−/−^/ApoE^−/−^ or their ApoE^−/−^ counterparts, aged between 10 and 12 weeks, were randomly assigned and fed for 10 weeks with a CED (21% fat, 0.15% cholesterol, 19.5% casein; Western‐type diet, Altromin GmbH, Lage, Germany) or continued to receive SD (LASQCdiet® Rod16‐R, LASvendi, Soest, Germany) as a control group. The precise composition of the diets is provided in Supporting Information S1. The animals were housed under pathogen‐free conditions in a 12‐h dark/12‐h light cycle and had access to food and water ad libitum. Genotyping and tissue harvesting were performed as previously described.^22^, ^23^ In brief, following the predefined feeding period, animals were anesthetized with a combination of ketamine (150 mg/kg) and xylazine (20 mg/kg). Euthanasia was performed at 20 weeks of age for CED‐fed genotypes and at 30 weeks for SD‐fed genotypes by surgical exposure of the heart and subsequent perfusion via apical access with phosphate‐buffered saline (PBS) containing 5 U/mL heparin (Liquemin® 25,000 U/5 mL, Roche, Grenzach, Germany), followed by 4% paraformaldehyde (PFA) in PBS. After perfusion, hearts were post‐fixed overnight in 4% PFA/PBS and subsequently embedded in paraffin (Sakura Finetek Europe, Alphen aan den Rijn, the Netherlands).

All experiments using animals were approved by the regional governmental animal ethics board (Regierungspräsidium Gießen, AZ V54‐19 c 2015 h 01 MR 20/26 Nr. 21/2014) and were conducted in accordance with the ARRIVE guidelines and with regulations governing animal studies at the Philipps University Marburg.

### 2.2 Determination of plasma lipid values

Blood was collected during right atrial incision and immediately mixed with heparin (0.25 I.U./mL; Roche) to prevent coagulation. Following centrifugation at 650*g* for 10 min, the resulting plasma was carefully separated and stored at −80°C until further use. Plasma cholesterol and triglyceride concentrations were subsequently measured by spectrophotometry using a microplate reader (Sunrise; Tecan, Männedorf, Switzerland) together with commercially available assay kits (Cholesterol/Cholesteryl Ester Quantitation Kit ab65359 and Triglyceride Quantification Assay Kit ab65336; Abcam, Cambridge, UK), according to the manufacturers' instructions.

### 2.3 Histological processing and staining

Formaldehyde‐fixed and paraffin‐embedded specimens were cut into 7 μm thin sections, and Giemsa and hematoxylin–eosin (H&E) staining were performed, as previously described.^24^ Picrosirius red (Catalogue No. VWR640745, VWR, Radnor, PA, USA) staining was used according to the manufacturer's protocol.

### 2.4 Qualitative and quantitative image analyses

After histochemical processing of the tissue sections, images were acquired at different magnifications (×20, ×100, ×200, ×400) using Zeiss Axio Imager.M2 (Zeiss, Oberkochen, Germany). All analyses were performed by investigators blinded to animal ID and genotype.

To measure the diameters of the ventricular walls, H&E‐stained sections sliced axially along the midventricular plane were scanned. The diameters of the septal and lateral walls of the left ventricle and the lateral wall of the right ventricle were measured with their axes aligned using the biological‐image analysis software Fiji.^25^ To account for potential dehiscence or cutting artifacts of the tissue or potential differences in body dimensions of the animals, each diameter was indexed to the sum of all diameters obtained from the corresponding section (e.g., left ventricular lateral wall diameter indexed = left ventricular lateral wall diameter/[left ventricular lateral wall diameter + septal wall diameter + right ventricular lateral wall diameter]).

To measure the cross‐sectional diameters of cardiomyocytes, images of H&E‐stained sections from three different locations (left lateral wall, septal wall, right lateral wall) were acquired with ×400 magnification. Subsequently, the cardiomyocytic cross‐sectional diameter was determined using Fiji and averaged for each animal.

To quantify fibrosis, images of picrosirius red (PSR)‐stained sections from three different myocardial regions (left lateral wall, septal wall, right lateral wall) were acquired at ×200 magnification. For each heart, one representative section per region was selected, resulting in three images per animal. This anatomically predefined sampling ensured consistent coverage across the left and right ventricles and the interventricular septum, while maintaining feasibility across the entire animal cohort. Image analysis was performed using the Fiji software. Color channels were separated using a custom ImageJ macro based on color deconvolution, which is provided in Supporting Information S2. The macro was specifically optimized to minimize erroneous signal from PSR‐stained cardiomyocyte nuclei. The red channel was then converted to binary values for quantification. To calculate the fibrotic area, the binary red channel values were divided by the total pixel count of the original image, yielding the percentage of fibrotic area relative to the total section area. Quantification was performed in a fully blinded fashion for all animals.

### 2.5 Statistical analyses

Statistical analyses were performed using GraphPad Prism 5.0 (GraphPad Software, La Jolla, CA, USA), with one‐way ANOVA and Bonferroni's multiple comparison test to assess statistical differences between groups after testing for normal distribution using the Kolmogorov–Smirnov test. Non‐normally distributed variables were compared using the Kruskal–Wallis test. A *p*‐value of <.05 was considered statistically significant. The number of animals analyzed per group (*n*) is indicated in the corresponding bar graphs and figure legends.

## 3 RESULTS

### 3.1 Analyses of macroscopic and cellular myocardial dimensions

**FIGURE 1:**
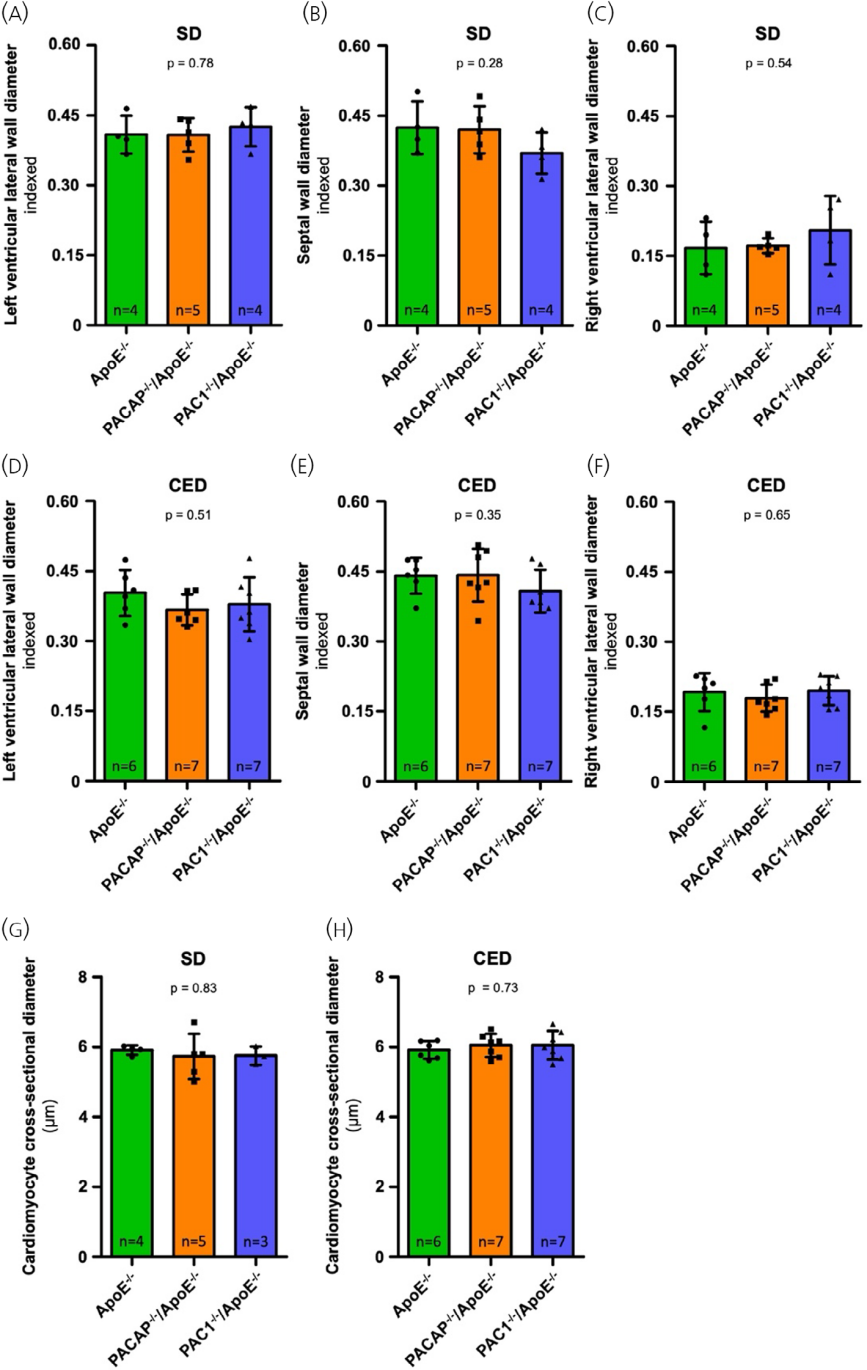
Analysis of ventricular wall dimensions and cardiomyocyte cross‐sectional diameter of mice fed with a standard chow diet (SD) (A–C, G) and cholesterol‐enriched diet (CED) (D–F, H). Under SD feeding conditions no statistically significant differences in the indexed dimensions of the left ventricular lateral wall (A), the septal wall (B) nor the right ventricular lateral wall (C) were observed among the genotypes studied. Also, under CED feeding conditions no statistically significant differences in the indexed dimensions of the left ventricular lateral wall (D), the septal wall (E), and the right ventricular lateral wall (F) were observed among the genotypes studied. Regarding the cardiomyocyte cross‐sectional diameter, no statistically significant differences were observed under either SD or CED feeding conditions among the genotypes studied (SD feeding: G; CED feeding: H). The quantitative data for the graphs shown can be found in Tables 1 and 2, respectively. Graphs display mean values and standard deviation. *n*—number of animals analyzed to generate the corresponding data.

**FIGURE 2:**
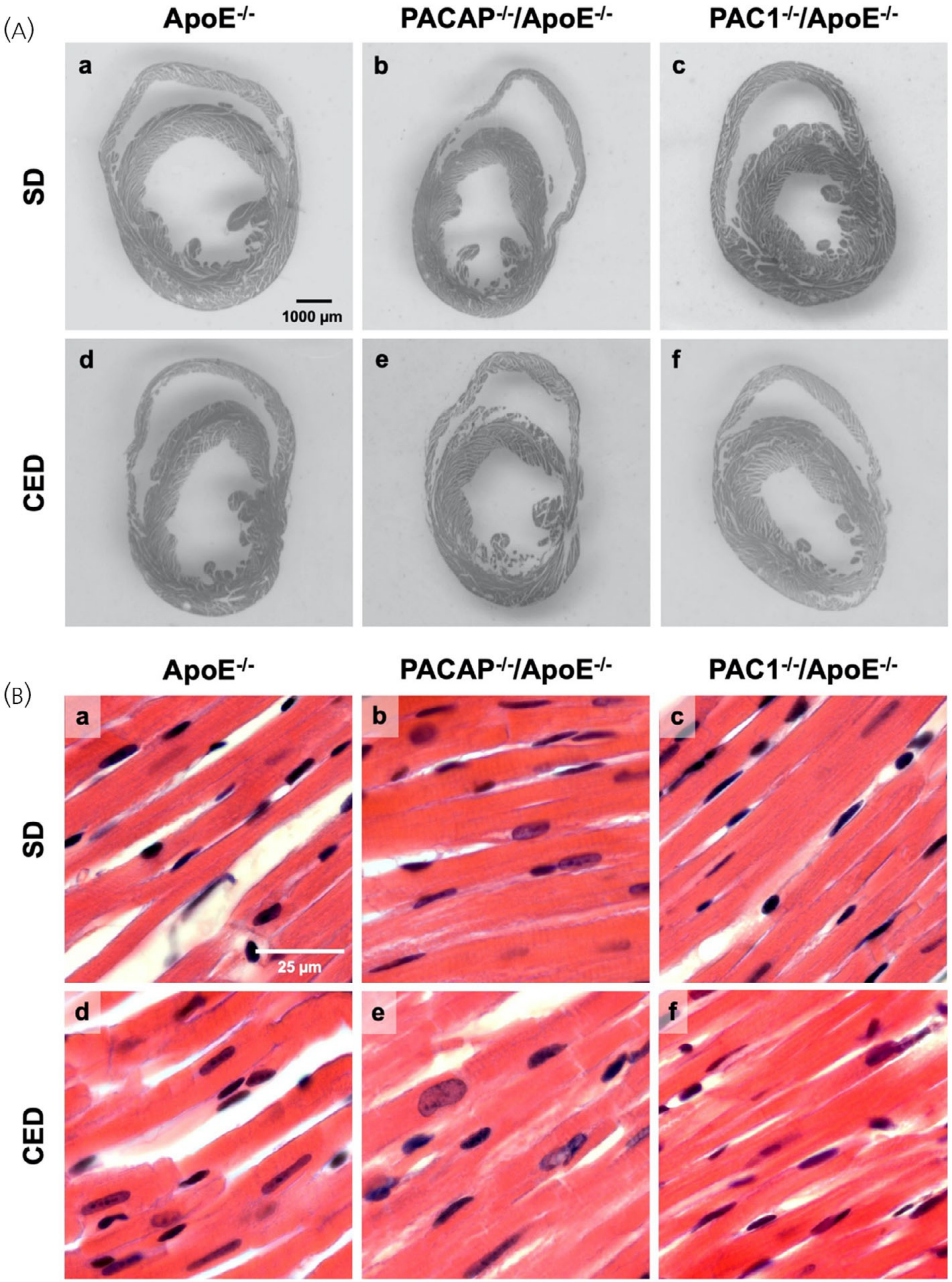
Macroscopic (A) and cellular (B) ventricular architecture. Subfigure (A) shows representative images of axial cardiac sections in the midventricular plane, demonstrating no relevant differences for the three genotypes studied (ApoE^−/−^, a, d; PACAP^−/−^/ApoE^−/−^, b, e; PAC1^−/−^/ApoE^−/−^, c, f) under SD (a–c) nor cholesterol‐enriched diet (CED) feeding conditions (d–f). Subfigure (B) shows representative images of H&E‐stained microscopic myocardial sections at ×400 magnification, also demonstrating no relevant differences for the three genotypes studied (ApoE^−/−^, a, d; PACAP^−/−^/ApoE^−/−^, b, e; PAC1^−/−^/ApoE^−/−^, c, f) under SD (a–c) or CED feeding conditions (d–f).

To test whether constitutional deficiency of PACAP or PAC1 has effects on cardiac architecture, morphometric analyses at the macroscopic and cellular levels were performed in ApoE‐deficient mice and in ApoE‐deficient mice in which the PACAP (Adcyap1) or PAC1 (Adcyap1r1) genes were deleted, after long‐term feeding with cholesterol‐rich (CED) or regular (SD) chow. There were no statistically significant differences in the indexed diameters of the lateral and septal walls of the left ventricle, nor in the lateral wall of the right ventricle between PACAP^−/−^/ApoE^−/−^, PAC1^−/−^/ApoE^−/−^ or ApoE^−/−^ mice under either SD or 10‐week CED. Figure 1A–F and Table 1 provide the corresponding quantitative data and Figure 2A shows representative cross‐sections of the respective genotypes and diets.

**TABLE 1:** Ventricular wall dimensions.

Diet	Ventricular region	Genotype			*p*‐value
		ApoE^−/−^	PACAP^−/−^/ApoE^−/−^	PAC1^−/−^/ApoE^−/−^	
Standard chow (SD)	LV lateral wall	0.409 ± 0.041	0.408 ± 0.036	0.425 ± 0.042	.76
	Septal wall	0.424 ± 0.057	0.420 ± 0.050	0.370 ± 0.044	.28
	RV lateral wall	0.167 ± 0.056	0.172 ± 0.016	0.205 ± 0.073	.55
Cholesterol‐enriched diet (CED)	LV lateral wall	0.403 ± 0.049	0.367 ± 0.033	0.379 ± 0.058	.44
	Septal wall	0.441 ± 0.039	0.442 ± 0.057	0.408 ± 0.046	.35
	RV lateral wall	0.192 ± 0.041	0.179 ± 0.031	0.195 ± 0.031	.65

*Note*: Quantitative data on indexed diameters of the corresponding ventricular walls of the genotypes and feedings studied. Data represent the mean and standard deviation.

Also, at the cellular level, no statistically significant differences in cardiomyocyte dimensions were detected between the studied genotypes or the corresponding diets, as displayed in Figure 1G,H, Table 2, and Figure 2B, respectively. Thus, in summary, the results show neither macroscopic nor cellular hyper‐ or hypotrophy in PACAP or PAC1 deficiency alone or under a Western‐type diet.

**TABLE 2:** Cardiomyocyte cross‐sectional diameters.

Diet	Genotype			*p*‐value
	ApoE^−/−^	PACAP^−/−^/ApoE^−/−^	PAC1^−/−^/ApoE^−/−^	
Standard chow (SD)	5.91 ± 0.13 μm	5.73 ± 0.65 μm	5.76 ± 0.26 μm	.83
Cholesterol‐enriched diet (CED)	5.92 ± 0.25 μm	6.05 ± 0.33 μm	6.05 ± 0.41 μm	.73

*Note*: Quantitative data on cardiomyocyte cross‐sectional diameter of the genotypes and feedings studied. Data represent the mean and standard deviation.

### 3.2 Analysis of myocardial fibrosis

The central objective of this present study was to analyze the effects of a lack of PACAP or PAC1 in the ApoE model on myocardial fibrosis. We compared results from mice under the two different feeding regimens.

**FIGURE 3:**
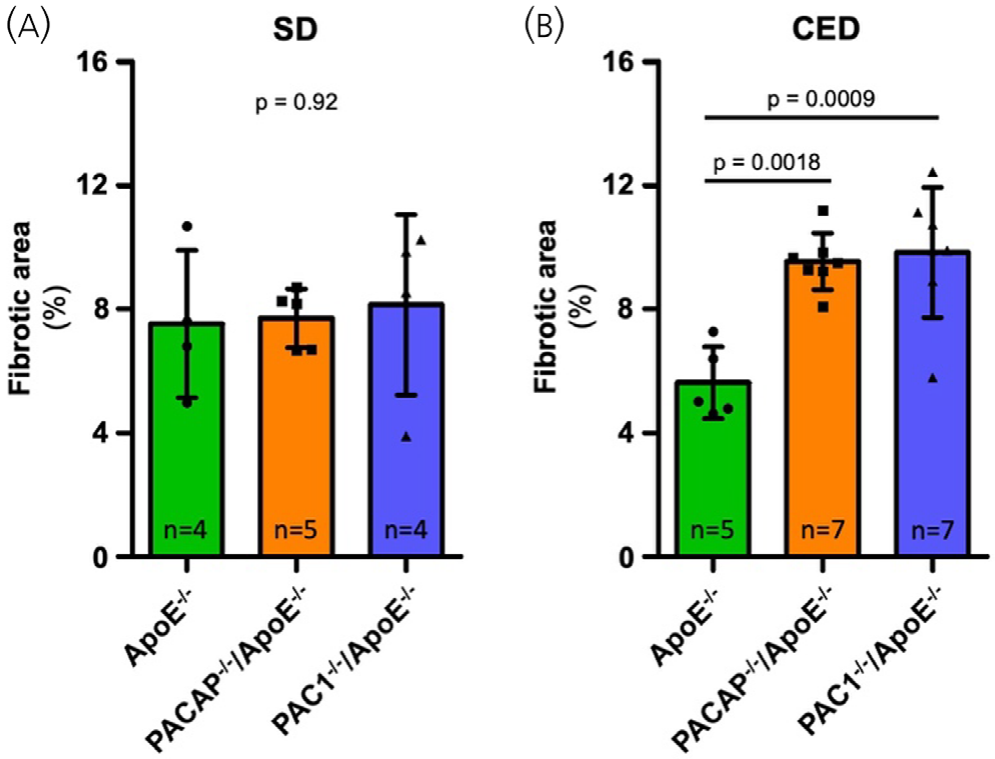
Analysis of fibrotic area in the myocardium under standard chow diet (SD) feeding conditions (A) and Western‐type cholesterol‐enriched diet (CED) feeding conditions (B). Under SD feeding conditions, no statistically significant differences in fibrotic area of the myocardium were observed among the genotypes studied (A; ApoE^−/−^ 7.5 ± 2.4%; PACAP^−/−^/ApoE^−/−^ 7.7 ± 0.9%; PAC1^−/−^/ApoE^−/−^ 8.1 ± 2.9%; *p* > .05). However, under CED feeding conditions, a statistically significant greater fibrotic area was observed in PACAP^−/−^/ApoE^−/−^, as well as in PAC1^−/−^/ApoE^−/−^, compared to ApoE^−/−^ mice (B; ApoE^−/−^ 5.6 ± 1.2%; PACAP^−/−^/ApoE^−/^ 9.5 ± 0.9%; PAC1^−/−^/ApoE^−/−^ 9.8 ± 2.1%; *p* < .001). Fibrotic area represents the percentage of fibrotic area to the total area of the histologic section. Graphs display mean values and standard deviation. *n*—number of animals analyzed to generate the corresponding data.

**FIGURE 4:**
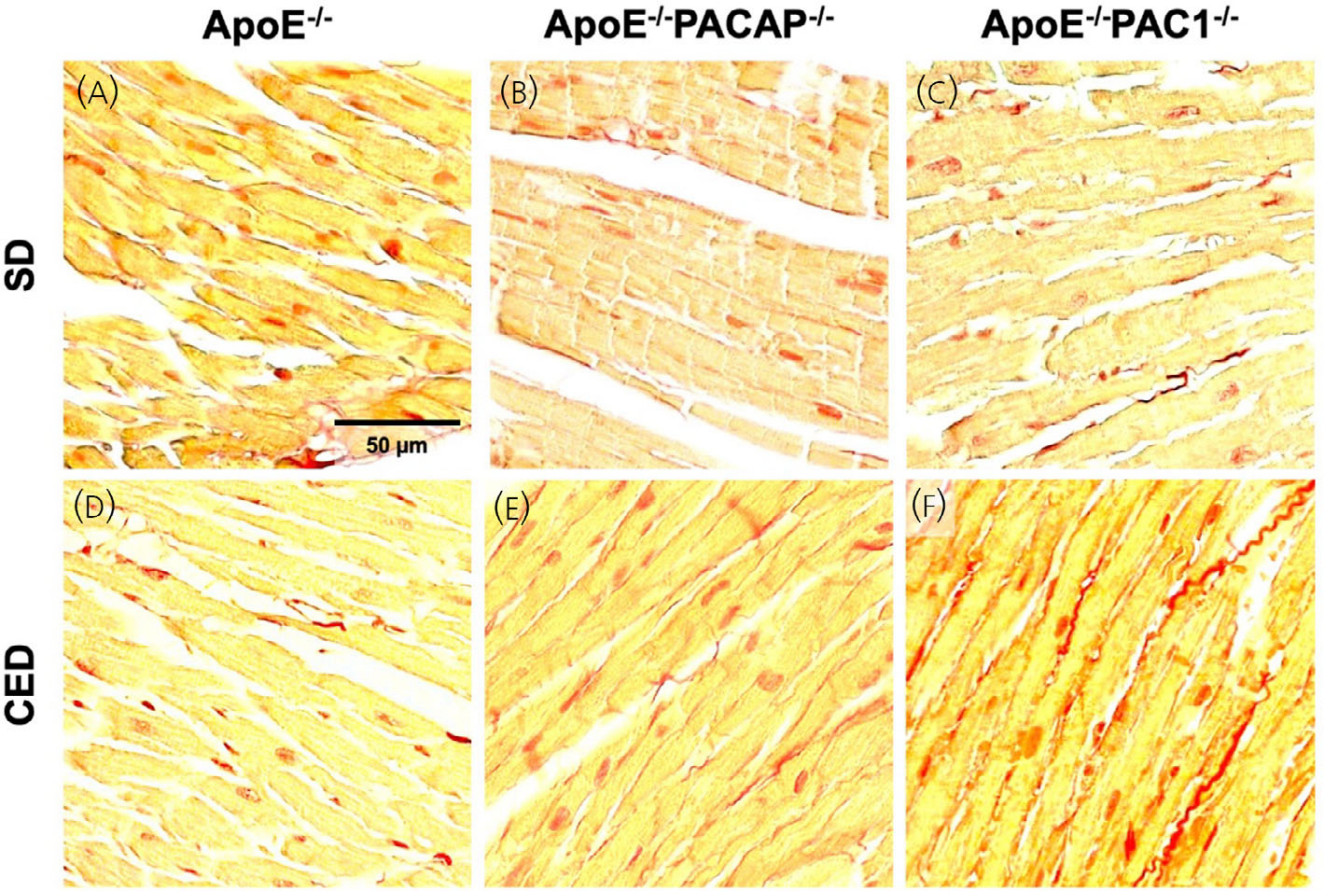
Myocardial fibrosis. Representative images of picrosirius red stained microscopic myocardial sections at ×200 magnification for the three genotypes studied (ApoE^−/−^, A, D; PACAP^−/−^/ApoE^−/−^, B, E; PAC1^−/−^/ApoE^−/−^, C, F) under standard chow diet (SD) (A–C) and cholesterol‐enriched diet (CED) feeding conditions (D–F). Comparatively, considerably more stained extracellular matrix is evident in PACAP^−/−^/ApoE^−/−^ (E) and PAC1^−/−^/ApoE^−/−^ (F) under CED feeding.

No statistically significant differences in myocardial fibrosis grade and distribution pattern could be detected under SD feeding. However, after 10 weeks of CED, a statistically significant increase in myocardial fibrosis was detected in PACAP^−/−^/ApoE^−/−^ and PAC1^−/−^/ApoE^−/−^, compared to ApoE^−/−^ mice. The pattern of fibrous deposition was consistent with that of reactive interstitial fibrosis and affected both ventricles uniformly. Figure 3 provides the corresponding quantitative data and Figure 4 shows representative picrosirius red stains of the respective genotypes and diets.

### 3.3 Analyses of plasma lipid composition

To verify the metabolic response to dietary intervention and to evaluate whether the observed effects of PACAP or PAC1 deficiency on myocardial fibrosis might be related to systemic lipid profiles, plasma concentrations of total cholesterol and triglycerides were measured. Neither under SD nor CED conditions were significant differences in plasma cholesterol or triglyceride levels detected between PACAP^−^/^−^/ApoE^−^/^−^, PAC1^−^/^−^/ApoE^−^/^−^, and ApoE^−^/^−^ mice. In contrast, comparison between dietary regimens revealed significantly higher plasma cholesterol concentrations in all genotypes fed CED compared with those on SD. With respect to triglycerides, a significant increase under CED compared with SD was observed only in ApoE^−^/^−^ mice. The corresponding plasma cholesterol and triglyceride values for each genotype and diet are summarized in Table 3.

**TABLE 3:** Plasma cholesterol and triglyceride levels.

Genotype	Cholesterol Plasma Level (mg/dl)		*p*‐value*	Triglyceride Plasma Level (mg/dl)		*p*‐value*
	SD	CED		SD	CED	
ApoE^−/−^	527.4 ± 32.5 (6)	1064.3 ± 73.2 (6)	<.001	96.0 ± 9.4 (5)	149.0 ± 10.7 (6)	<.01
PACAP^−/−^/ApoE^−/−^	638.6 ± 44.8 (5)	1031.4 ± 70.0 (7)	<.01	127.3 ± 24.2 (6)	187.2 ± 37.2 (8)	n.s.
PAC1^−/−^/ApoE^−/−^	482.4 ± 58.9 (4)	1030.9 ± 55.4 (7)	<.001	105.8 ± 18.7 (5)	222.4 ± 45.2 (7)	n.s.
*p*‐value^#^	n.s.	n.s.		n.s.	n.s.	

*Note*: The data show means ± standard error of the mean. Statistical comparisons were performed using the Kruskal–Wallis test; *n* indicates number of animals.

Abbreviations: ApoE, apolipoprotein E; CED, cholesterol‐enriched diet; n.s., not significant; PACAP, pituitary adenylate cyclase‐activating polypeptide; PAC1, pituitary adenylate cyclase‐activating polypeptide type I receptor; SD, standard chow diet.

* indicates the *p*‐value for comparisons between diets; # indicates the *p*‐value for comparisons between genotypes.

## 4 DISCUSSION

Deletion of PACAP expression in ApoE knockout mice markedly accelerated interstitial cardiac fibrosis, under CED‐induced metabolic stress conditions. The absence of fibrosis and preservation of cardiac macroscopic and cellular architecture under SD feeding conditions suggest that there is no obvious perturbation of cardiac development in PACAP‐deficient mice. Deletion of PACAP (Adcyap1) in ApoE knockout mice also had no apparent effect on macroscopic and cellular cardiac architecture under either the SD or Western‐type CED. Furthermore, the deficiency of PACAP in the ApoE^−/−^ background had no significant effect on cholesterol uptake in the time frame of feeding used in this study. This indicates—while confirming that CED feeding effectively induced a metabolic stress environment—that cardiac fibrosis appears to be influenced by PACAP–PAC1 signaling primarily through its modulatory effects on inflammation rather than lipid metabolism itself.

PACAP signaling can occur under various physiological conditions via activation of its principal receptor, PAC1, or the related receptors VPAC1 and VPAC2. We therefore examined whether or not PAC1‐deficient mice would share the PACAP‐deficient phenotype associated with progressive cardiac fibrosis in HFD‐fed ApoE^−/−^ mice. Deletion of PAC1 (Adcyap1r1), similarly to deletion of PACAP, in ApoE knockout mice had no effect on cellular cardiac architecture under either the SD or Western‐type CED, or on cholesterol uptake throughout the course of the study. As in PACAP‐deficient mice, there was no obvious perturbation of cardiac development in PAC1‐deficient mice. PAC1 deletion in HFD‐fed ApoE knockout mice phenocopied the major pathophysiological consequence of the deletion of PACAP; however, it accelerated interstitial cardiac fibrosis in these mice. Thus, the antifibrotic effect of PACAP is likely to occur via signaling through the PAC1 receptor. When interpreting these data, it is essential to consider the design‐related differences between the dietary regimens. Although a direct comparison between SD‐ and CED‐fed animals might seem informative, such an analysis would be confounded by the differences in total feeding duration and animal age (30 weeks for SD vs. 20 weeks for CED). ApoE^−^/^−^ mice are known to exhibit age‐dependent fibrotic remodeling,^26^ and the nonlinear relationship between exposure time and fibrotic progression precludes a cross‐diet comparison. Therefore, we focused on the primary objective of our study—the genotype‐dependent effects within each metabolic condition—to ensure biological validity and interpretative clarity.

Given the vasodilatory profile of endogenous PACAP signaling, PACAP or PAC1 deficiency could result in perturbations of cardiovascular hemodynamics leading to hypertension.^27^ However, the absence of signs of macroscopic and cellular cardiac hypertrophy, as demonstrated in Figures 1 and 2, which show unaltered ventricular wall diameters and cardiomyocyte diameters, makes this possibility unlikely. Our findings are in accord with previous reports that cardiomyocyte dimensions are unchanged in PACAP‐deficient mice.^14^, ^28^ Therefore, the deleterious effects of PACAP and PAC1 deficiency are unlikely to be caused by hypertension‐induced hypertrophic fibrotic remodeling. Taken together, these data suggest that the absence of PACAP or its primary receptor PAC1, exacerbates cardiac fibrosis not as a primary consequence of altered cardiovascular physiology, but rather secondary to the absence of anti‐inflammatory modulation during high‐fat diet ingestion, an inflammatory chronic event.

As compared to SD feeding conditions, CED feeding conditions for a duration of 10 weeks do not result in significantly higher fibrosis in ApoE^−/−^ mice.^28^, ^29^ However, feeding a CED regimen for as little as 4–10 weeks has been shown to cause significant systemic and tissue inflammation in ApoE^−/−^ mice.^29^, ^30^ In light of these previous findings, the markedly increased fibrosis in PACAP‐ and PAC1‐deficient ApoE knockout mice compared to ApoE^−/−^/PACAP^+/+^/PAC1^+/+^ reported here to occur even under short‐term CED implies that the PACAP–PAC1 axis acts as an early and effective anti‐inflammatory and anti‐fibrotic signaling pathway. This is in line with various reports of anti‐inflammatory and anti‐apoptotic effects of PACAP and PAC1 signaling in the context of atherosclerogenesis and cardiac damage associated with chemotherapeutic agents.^14^, ^15^, ^16^, ^22^, ^31^, ^32^

The mechanisms of the pro‐fibrotic effect of PACAP and PAC1 deficiency revealed by our study remain to be determined. It is reasonable to assume that cardiac fibroblasts and their activated forms, myofibroblasts, are involved in mediating the effects of the PACAP–PAC1 axis, since these represent essential regulators of cardiac ECM homeostasis and conductors of the cardiac response to stressors.^33^, ^34^ This is supported by the expression of the PAC1 receptor on fibroblasts, as shown by data from the human protein atlas^35^ (www.proteinatlas.org), and the downregulation of protein synthesis induced by PACAP in cultured fibroblasts.^13^ PACAP has also been shown to increase the synthesis and secretion of ANP in cultured cardiomyocytes.^13^ ANP, in turn, inhibits transformation and proliferation of cardiac fibroblasts and reduces expression of ECM molecules in myofibroblasts via inhibition of transforming growth factor beta (TGF‐β) signaling.^36^ An in vivo murine model of diabetic nephropathy has shown an anti‐fibrotic effect of PACAP treatment, resulting in the downregulation of collagen IV and TGF‐β synthesis in renal tissue.^37^

Evidence of a protective effect of PACAP and PAC1 signaling on cardiac structure and function exists primarily in models of cardiotoxicity or ischemia and reperfusion.^38^ Most consistently, the reduction of oxidative stress resulting in an anti‐apoptotic effect of PACAP on cardiomyocytes has been described,^14^, ^16^, ^31^, ^32^, ^39^ with corresponding anti‐fibrotic and anti‐inflammatory consequences. These effects of PACAP may be secondary to the induction of other anti‐inflammatory mediators with primary effects on cardiac tissues, such as selenoproteins inducible by PACAP.^40^ Indeed, cardiomyopathy is often triggered by inflammatory and immunometabolic conditions, for example, diabetes,^41^ in which PACAP may play a role.

There is not much evidence to date linking cardio‐protective effects to PACAP signaling in humans. A small‐case study described reduced PACAP‐38‐like immunoreactivity and lower PAC1 expression in tissue samples from patients with chronic heart failure.^42^ Impaired cardiac function and alterations in ventricular architecture, as seen in the cited cohort, have been firmly linked to fibrotic remodeling of the myocardium.^3^, ^34^ This indicates that PACAP may also serve a role in the pathology of human cardiac fibrosis. The conservation of PACAP and PAC1 structure and function at both the molecular and cellular levels, and the clinical trials that have already been performed (e.g., NCT04197349, NCT03238781) based on results achieved in the murine system, underlines the translational relevance of our data. The pleiotropic nature and the complex involvement of PACAP–PAC1 signaling at different stages of inflammatory disease^22^, ^23^, ^43^ also suggest that further translation in the context of HFpEF should proceed with a clear definition of the treatment regimen and expected clinical outcomes. In this regard, the ApoE atherosclerosis/cardiac fibrosis models offer a robust platform for the potential development of PACAP‐biased agonists, which may offer benefit in multiple aspects of cardiac disease. Currently ongoing efforts to better understand the multidimensionality of PACAP signaling through the PAC1 receptor, and the development of small molecule therapeutics that precisely target specific variants of the PAC1 receptor, will hopefully help to overcome these hurdles.^44^, ^45^, ^46^ Thus, it is of particular interest to have not only identified a potential antifibrotic effect for which a PAC1 agonist may be of therapeutic benefit. In fact, PACAP (PAC1) agonism may be useful in cardiovascular therapy particularly in approaching PACAP's role in cardiovascular function in the periphery separately from its well‐documented role in central regulation of stress responding.^47^, ^48^

### 4.1 Limitations

The present study does not provide further information on molecular mechanisms of the findings presented. Furthermore, we cannot conclude any effects of the experimental setup on cardiac and hemodynamic functions as functional data were not gathered. Nevertheless, a highly relevant clinical readout – cardiac fibrosis – was addressed. Another limitation arises from the difference in total feeding duration and age of the animals between the SD and CED groups (30 weeks and 20 weeks, respectively). Since myocardial fibrosis in ApoE^−^/^−^ mice develops in an age‐dependent, non‐linear manner, a direct comparison of the effects between the dietary regimes is not conclusive. Therefore, all analyses were performed separately within each dietary condition. Additionally, wall thickness and chamber dimensions were derived from fixed hearts, potentially introducing variability due to differences in cardiac relaxation. However, such effects would affect all groups equally and are unlikely to bias group comparisons. Similarly, fibrosis quantification was based on three anatomically defined myocardial regions per heart. While whole‐heart analysis would provide greater spatial resolution, this was beyond the logistical scope of the study. Finally, although our image analysis algorithm was optimized to exclude nuclear artifacts, minimal residual signal may have influenced fibrotic tissue area values. Again, any such effect would represent a systematic error and would not compromise the validity of intergroup comparisons.

## 5 CONCLUSION AND FUTURE DIRECTIONS

We provide new evidence for a potentially clinically relevant role of PACAP–PAC1 signaling in metabolically induced cardiac fibrosis. The antifibrotic effect of PACAP suggested by our results is considerable, is likely mediated via the major (PAC1) receptor for PACAP, and appears to emerge only under metabolic stress conditions caused by CED. The absence of macroscopic and cellular cardiac hypertrophy in PACAP‐deficient or PAC1‐deficient mice, as shown here, precludes relevant hemodynamic effects in the mechanism of PACAP–PAC1 signaling in the context of cardiac fibrosis. The findings of our study give reason to warn of potential chronic and detrimental cardiac side effects of PAC1 antagonists, such as those currently being tested, for the treatment of migraine.

According to our results, PAC1 receptor agonists, particularly if biased towards anti‐fibrotic signaling, could hold therapeutic potential to attenuate metabolically triggered cardiac fibrosis and would represent a much‐needed therapeutic option for the treatment of this adverse tissue remodeling, occurring frequently in cardiac disease. Future studies should include longitudinal designs with serial cardiac phenotyping and multimodal functional and molecular readouts to further validate the translational relevance of PACAP–PAC1 signaling in metabolic cardiac remodeling.

## AUTHOR CONTRIBUTIONS

**J. M. Schubart:** Investigation. **M. K. H. Schaefer:** Investigation. **G. A. Bonaterra:** Investigation. **L. Mey:** Investigation. **H. Schwarzbach:** Investigation. **S. Pankuweit:** Investigation. **F. Ausbuettel:** Investigation. **L. E. Eiden:** Writing – original draft; writing – review and editing; data curation; resources. **S. Weyand:** Investigation. **E. Weihe:** Conceptualization; writing – review and editing; writing – original draft; data curation; project administration; supervision. **R. Kinscherf:** Conceptualization; investigation; funding acquisition; writing – original draft; methodology; validation; visualization; writing – review and editing; project administration; data curation; supervision; resources. **C. Waechter:** Conceptualization; investigation; writing – original draft; funding acquisition; methodology; validation; visualization; writing – review and editing; software; formal analysis; data curation.

## FUNDING INFORMATION

This research was supported in part by the von Behring‐Röntgen‐Stiftung, Marburg (vBR 62‐0003) and partly by the Clinician Scientist Program (SUCCESS) of the Medical Faculty of the Philipps University Marburg, Germany. PACAP‐deficient mice were generated with support from NIMH‐IRP, MH002386. Open Access funding provided by the Open Access Publishing Fund of Philipps‐Universität Marburg with support from the Deutsche Forschungsgemeinschaft (DFG, German Research Foundation).

## CONFLICT OF INTEREST STATEMENT

The authors declare no conflicts of interests.

## Supporting information


**Data S1.** Supporting Information.

## Data Availability

The datasets generated and analyzed during the current study are available from the corresponding author upon reasonable request. All relevant data supporting the findings of this study are included in the manuscript and its supplementary materials. No publicly available datasets were used or generated.
