# Supplementary material for: Cardiac fibrosis induced by high‐fat diet in ApoE‐deficient male mice is exacerbated by genetic deletion of PACAP–PAC1 signaling
Source: J Neuroendocrinol. 2025 Nov 25;38(1):e70118. doi: 10.1111/jne.70118 (PMC12799326; doi:10.1111/jne.70118)

**Cardiac fibrosis induced by high fat diet in ApoE-deficient male mice is exacerbated by genetic deletion of PACAP-PAC1 signaling**

Schubart JM et al.

**Supplementary Material S1** – Composition of the diets

**Standard Chow Diet (LASQCdiet® Rod16-R, LASvendi, Soest, Germany)**

### Main Nutrients (per kg)
- **Protein:** 16.9%
- **Fat:** 4.3%
- **Fiber:** 4.3%
- **Ash:** 7.0%
- **Nitrogen-Free Extracts:** 55.5%
- **Dry Matter:** 88.0%

### Energy Content (per kg)
- **Gross Energy (GE):** 15.9 MJ
- **Metabolizable Energy (ME):** 13.1 MJ (Estimated by §14 FMVO)

### Minerals (per kg)
- **Calcium:** 10.0 g
- **Phosphorus:** 6.5 g
- **Sodium:** 3.0 g
- **Magnesium:** 2.5 g

### Trace Elements (per kg)
- **Iron:** 200.0 mg
- **Iodine:** 4.0 mg
- **Copper:** 15.0 mg
- **Cobalt:** 1.5 mg
- **Manganese:** 120.0 mg
- **Selenium:** 0.2 mg
- **Zinc:** 75.0 mg

### Vitamins (per kg)
- **Vitamin A:** 15,000 IU
- **Vitamin D3:** 1,200 IU
- **Vitamin E:** 90 mg
- **Vitamin K:** 5 mg
- **Thiamine (B1):** 15 mg
- **Riboflavin (B2):** 10 mg
- **Pyridoxine (B6):** 10 mg
- **Cobalamin (B12):** 50 mg
- **Biotin:** 200 mcg
- **Choline Chloride:** 1,000 mg
- **Folic Acid:** 2 mg
- **Niacin:** 40 mg
- **Pantothenic Acid:** 20 mg

### Fatty Acids (per kg)
- **C16:0:** 5.0 g
- **C18:0:** 2.0 g
- **C20:0:** 0.1 g
- **C18:1:** 9.0 g
- **C18:2:** 19.0 g
- **C18:3:** 7.5 g

**Western Type Diet (21% fat, 0.15% cholesterol, 19.5% casein; Western Type diet, Altromin GmbH, Lage, Germany)**

## ### Metabolized Energy Content

- **Fat:** 1,898 kcal/kg (43%)
- **Protein:** 1,252 kcal/kg (28%)
- **Carbohydrates:** 1,282 kcal/kg (29%)

## ### Crude Nutrients and Moisture

- **Moisture:** 71,068 mg/kg (7.1%)
- **Crude Ash:** 56,377 mg/kg (5.6%)
- **Crude Fibre:** 19,037 mg/kg (1.9%)
- **Crude Fat:** 210,914 mg/kg (21.1%)

- **Crude Protein:** 312,906 mg/kg (31.3%)
- **Nitrogen-Free Extract:** 329,698 mg/kg (33%)

## ### Carbohydrates

- **Monosaccharides:** 28,973 mg/kg
- **Disaccharides:** 16,623 mg/kg
- **Polysaccharides:** 195,942 mg/kg

## ### Minerals

- **Calcium:** 8,440 mg/kg
- **Potassium:** 3,152 mg/kg
- **Magnesium:** 1,031 mg/kg
- **Sodium:** 4,037 mg/kg
- **Phosphorus:** 4,930 mg/kg

## ### Trace Elements

- **Aluminium:** 143.40 mg/kg
- **Chlorine:** 6,623.46 mg/kg
- **Iron:** 120.38 mg/kg
- **Fluorine:** 5.78 mg/kg
- **Iodine:** 1.28 mg/kg
- **Cobalt:** 0.53 mg/kg
- **Copper:** 8.93 mg/kg
- **Manganese:** 34.53 mg/kg
- **Molybdenum:** 0.39 mg/kg
- **Sulfur:** 1,596.00 mg/kg
- **Selenium:** 0.44 mg/kg
- **Zinc:** 46.34 mg/kg

## ### Added Vitamins

- **Vitamin A:** 15,000 IU/kg
- **Vitamin D3:** 600 IU/kg
- **Vitamin E:** 94 mg/kg
- **Vitamin K3:** 3 mg/kg
- **Vitamin B1:** 18 mg/kg
- **Vitamin B2:** 12 mg/kg
- **Vitamin B6:** 9 mg/kg
- **Vitamin B12:** 35 µg/kg
- **Nicotinic Acid:** 36 mg/kg
- **Pantothenic Acid:** 21 mg/kg
- **Folic Acid:** 2 mg/kg
- **Biotin:** 300 µg/kg
- **Choline Chloride:** 833 mg/kg
- **Vitamin C:** 36 mg/kg

## ### Amino Acids

- **Alanine:** 10,183 mg/kg
- **Arginine:** 18,605 mg/kg
- **Aspartic Acid:** 13,281 mg/kg
- **Cystine:** 4,254 mg/kg
- **Glutamic Acid:** 45,831 mg/kg
- **Glycine:** 17,344 mg/kg
- **Histidine:** 7,298 mg/kg
- **Isoleucine:** 11,411 mg/kg
- **Leucine:** 20,469 mg/kg
- **Lysine:** 23,123 mg/kg
- **Methionine:** 8,875 mg/kg
- **Phenylalanine:** 11,911 mg/kg
- **Proline:** 24,914 mg/kg
- **Serine:** 10,895 mg/kg
- **Threonine:** 10,770 mg/kg
- **Tryptophan:** 2,908 mg/kg
- **Tyrosine:** 11,851 mg/kg
- **Valine:** 8,512 mg/kg

## ### Fatty Acids

- **Arachidic Acid C-20:0:** 1,340 mg/kg
- **Eicosanoic Acid C-20:1:** 1,675 mg/kg
- **Alpha-Linolenic Acid C-18:3:** 13,159 mg/kg

- **Linolenic Acid C-18:2:** 94,145 mg/kg
- **Palmitic Acid C-16:0:** 24,011 mg/kg
- **Stearic Acid C-18:0:** 9,089 mg/kg
- **Oleic Acid C-18:1:** 45,798 mg/kg

**Supplementary Material S2** – Macro code for image analysis

Fiji code for the macro used to separate color channels:

macro "fibrosis" {

rename("image.tif");

saveAs("Jpeg")

run("Duplicate...",  "title=image duplicate.tif");

run("Split Channels")

selectWindow("image.tif (green)");

close();

selectWindow("image.tif (blue)");

close();

selectWindow(„image duplicate.tif")

setOption("BlackBackground", false);

run("Make Binary")

run("Fill Holes");

run("Measure")

rename("tissue area")

saveAs("Jpeg");

close();

selectWindow("tissue.tif (red)")

rename("fibrotic area")

setOption("BlackBackground", false);

run("Make Binary");

run("Fill Holes");

run("Measure");

saveAs("Jpeg");

close();

selectWindow("Results")

saveAs("Results");

}

Example of the different images generated:


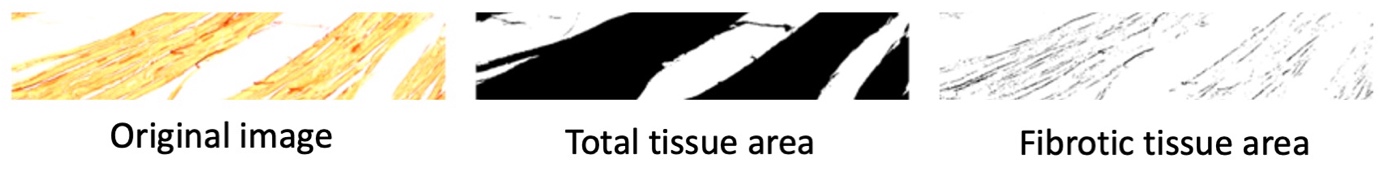

Supplement: Supplementary file 1 — Data S1. Supporting Information. [file JNE-38-e70118-s001.docx]
